# Surface-enhanced Raman spectroscopy of blood serum based on gold nanoparticles for the diagnosis of the oral squamous cell carcinoma

**DOI:** 10.1186/s12944-017-0465-y

**Published:** 2017-04-07

**Authors:** Yingyun Tan, Bing Yan, Lili Xue, Yi Li, Xianyang Luo, Ping Ji

**Affiliations:** 1 Stomatological Hospital of Chongqing Medical University, Chongqing, 400000 China; 2 Chongqing Key Laboratory of Oral Diseases and Biomedical Sciences, Chongqing, 400000 China; 3 Chongqing Municipal Key Laboratory of Oral Biomedical Engineering of Higher Education, Chongqing, 400000 China; 4 Department of Otolarygology Head and Neck Surgery, the First Affiliated Hospital of Xiamen University, Xiamen, 361000 China; 5 Department of Stomatology, the First Affiliated Hospital of Xiamen University, Xiamen, 361000 China; 6 Department of Head and Neck Oncology, the West China Hospital of Stomatology, Sichuan University, Chengdu, 610000 China

**Keywords:** Oral squamous cell carcinoma (OSCC), Surface-enhanced Raman spectroscopy (SERS), Principal component analysis (PCA), Linear discriminant analysis(LDA), Diagnosis

## Abstract

**Background:**

Oral squamous cell carcinoma (OSCC) is becoming more common across the globe. The prognosis of OSCC is largely dependent on the early detection. But the routine oral cavity examination may delay the diagnosis because the early oral malignant lesions may be clinically indistinguishable from benign or inflammatory diseases. In this study, the new diagnostic method is developed by using the surface enhanced Raman spectroscopy (SERS) to detect the serum samples from the cancer patients.

**Method:**

The blood serum samples were collected from the OSCC patients, MEC patients and the volunteers without OSCC or MEC. Gold nanoparticles(NPs) were then mixed in the serum samples to obtain the high quality SERS spectra. There were totally 135 spectra of OSCC, 90 spectra of mucoepidermoid carcinoma (MEC) and 145 spectra of normal control group, which were captured by SERS successfully. Compared with the normal control group, the Raman spectral differences exhibited in the spectra of OSCC and MEC groups, which were assigned to the nucleic acids, proteins and lipids. Based on these spectral differences and features, the algorithms of principal component analysis(PCA) and linear discriminant analysis (LDA) were employed to analyze and classify the Raman spectra of different groups.

**Results:**

Compared with the normal groups, the major increased peaks in the OSCC and MEC groups were assigned to the molecular structures of the nucleic acids and proteins. And these different major peaks between the OSCC and MEC groups were assigned to the special molecular structures of the carotenoids and lipids. The PCA-LDA results demonstrated that OSCC could be discriminated successfully from the normal control groups with a sensitivity of 80.7% and a specificity of 84.1%. The process of the cross validation proved the results analyzed by PCA-LDA were reliable.

**Conclusion:**

The gold NPs were appropriate substances to capture the high-quality SERS spectra of the OSCC, MEC and normal serum samples. The results of this study confirm that SERS combined PCA-LDA had a giant capability to detect and diagnosis OSCC through the serum sample successfully.

## Background

Oral squamous cell carcinoma (OSCC) is among the 10th most common cancer in the world, and the annual incidence of OSCC continues to increase specifically in Western and Asian countries [1, 2]. It is reported 3.29 per 100,000 as incidence rate and 1.49 per 100,000 as mortality rate in China [3]. The survival of OSCC is poor, with a 5-years survival rate of approximately 50% and has not a remarkable improve in the recent decades. The early detection by screening can be considered the best method to improve survival [4]. However the oral cavity is accessible to physical examination, the clinical visual examinations occasionally may delay the diagnosis because the early oral malignant lesions may be clinically indistinguishable from benign or inflammatory diseases [5]. The reliability of visual examination is also questionable and there are insufficient evidence to recommend a visual examination in screening for oral cancer in a low-risk population [5, 6]. The oral biopsy is considered the golden standard of OSCC diagnosis, but it is time consuming, labor intensive and invasive [7]. So a real-time, accurate and non-invasive diagnostic method is a pressing need for OSCC detection.

The surface-enhanced Raman spectroscopy (SERS) was first discovered by Fleichman and his colleages who founded the Raman intensities were enhanced as much as 10^5^–10^14^ times by adsorbing molecules onto nanostructured metal surfaces [8–10]. SERS can overcome the drawbacks of regular Raman spectroscopy such as weak Raman intensities and strong autofluorescence background, and make the possibility of detecting trace or even single molecule. The SERS have been applied successfully on the detection of oncological diseases in different sites [11]. The key point of successful diagnosis by Raman spectroscopy is the dependence on the visual detection of morphologically or structurally aberrant lesions [2]. But the early oral carcinoma may be visually indistinguishable due to the subtle morphological alterations, and the Raman spectroscopic technique can not focus exactly on the lesions which could result in the false diagnosis. And some OSCC arising on the hidden sites in oral cavity such as the tongue base are not easy to be diagnosed at the early stage. So the spectral detection and analysis of biofluids using SERS is considered a novel, non-invasive and convenient method for the OSCC diagnosis. A systematic study based on the SERS of blood plasma and serum recommends the serum as the sample of choice due to the interference of anticoagulants in common plasma and various pathological metabolites in patients’ serums [12]. In our previous study, SERS combined with support vector machine has successfully distinguished the patients with different parotid gland tumors from the normal subjects by detecting the blood serums [10]. So in this study, we developed the method of blood serum detection by using SRES based on gold nanoparticles to diagnose the OSCC.

## Methods

### Subjects and protocol

In this study, a total of 135 patients with OSCC were selected as the experimental group, who were not treated prior to this study and didn’t have any other sysmetic diseases or drug abuse. In order to demonstrate the ability of discrimination of the method, 90 patinets with the mucoepidermoid carcinoma (MEC) were selected as the positive control group. And a total of 145 patients with old maxillofacial fracture and healthy volunteers were selected as the normal control group. More information on the subjects was shown in Table 1. At the beginning of the study, all subjects were informed detailedly and gave their written informed consents. The routine blood examinations of all subjects remained in the normal range. And this study was approved by the Ethics Committees of the First Affiliated Hospital of Xiamen University and followed the guidelines of the Helsinki Declaration.

**Table 1:** Information on these subjects in this study

Information	Group		
	OSCC	MEC	Normal
Age(year)			
Age rang	39–70	28–79	18–68
Median age	58	44	36
Gender			
Male	75	50	93
Female	60	40	52
Total	135	90	145

### Preparation of the gold nanoparticles

Gold nanoparticles(NPs) were prepared through the deoxidizing process reported in the our previous study [9]. A total of 0.7 ml of 1% *w*/*v* trisodiumcitric acid was added rapidly in the beaker of rolling boiled 100 ml HAuCl_4_. The mixed solution was heated to keep boiling and stirred continuously for half hours. The golden NPs were produced when the color of the solution changed from pale yellow to burgundy. The experimental conditions of different batches were controlled very carefully to guarantee the consistency of the shape and volume of the gold NPs. The gold NPs solution was stored at 4 °C for the SERS measurement.

### Preparation of blood serum samples

A total of 5 ml peripheral blood sample was obtained from the subject who had fasted overnight for 10 h. The obtained blood sample was deposited at 4 °C for 4 h without any anticoagulant. Then the blood sample was centrifuged at 3400 rpm for 10 mins in order to remove the cells, fibrinogen and platelet in the blood. A single 1 ml supernatant serum sample was collected from the centrifuged blood and stored at −20 °C the Raman detection.

### Scanning electron microscopy (SEM) and the ultraviolet-visible spectroscopy analysis

The morphologies of the gold NPs were detected by using a scanning electron microscope (HITACHI S-4800, Hitachi Ltd., Tokyo, Japan) with a voltage of 30 kV. The absorption of the gold NPs and the mixture of the serum and gold NPs were monitored by the ultraviolet-visible spectroscope (Cary5000, VARIAN Ltd., USA).

### SERS measurement

A total 4 ml of gold NPs solution prepared in the above processing was added into a tube and centrifuged at 6000 rpm for 10mins. Then the supernatant was discarded from the gold NPs and a 0.4 ml serum sample was added for the SERS measurement. The mixed solution was vibrated by ultrasonic oscillator in order to make the NPs distribute more homogeneously. The solution was incubated at 4 °C for 2hs before the SERS measurement. The SERS measurement was carried out by using a Renishaw inVia Raman microscope (Renishaw Ltd., UK) with a 633 nm laser. The excitation laser with a power of about 0.4 W was focused on the serum samples through a ×50 objective lens (NA = 0.75). The spectra were recorded in the 200–1800 cm^−1^ Raman shift range with a 2 cm^−1^ spectral resolution. Every spectrum was integrated for 10s and averaged over 2 accumulations.

### Data pre-processing

Before the data statistical analysis, the raw Raman spectral data were preprocessed by WiRE 2.0 software (Renishaw Ltd., UK) to remove the noisy interferences and oversaturated spectra. The autofluorescence backgrounds were removed by the 4th degree polynomial function and the SERE spectra were smoothed by the Savitzky-Golay smoothing through the LABSPEC 2.0 software (HORIBA Scientific, France). Then the baseline correction and normalization were carried out before the further analysis and comparison of the different spectra.

The mean spectra of different groups were obtained by calculating and analyzing the pre-processed data through the OringinPro 8.0 software (OringinLab, USA). The spectral differences between the groups were obtained by subtracting the mean spectra of different groups. The differences of peaks shown in the subtracted spectra were assigned to the molecular structures and biochemical component based on the results reported in the previous literatures.

### Multivariate analysis

Principal component analysis(PCA) was employed to reduce the dimensions and determine the key variables. In this study, the retaining principal components which accounted for 90% of the variance in the spectral data was applied as statistical variables and input into Linear discriminant analysis(LDA) for the serum classification. The prediction performance of the diagnostic model rendered by LDA was estimated and evaluated by the leave-one-out-cross-validation method. This study discriminated the OSCC group from the normal group firstly, and then discriminated the OSCC group from the MEC group and the normal group.

## Results

**Fig. 1:**
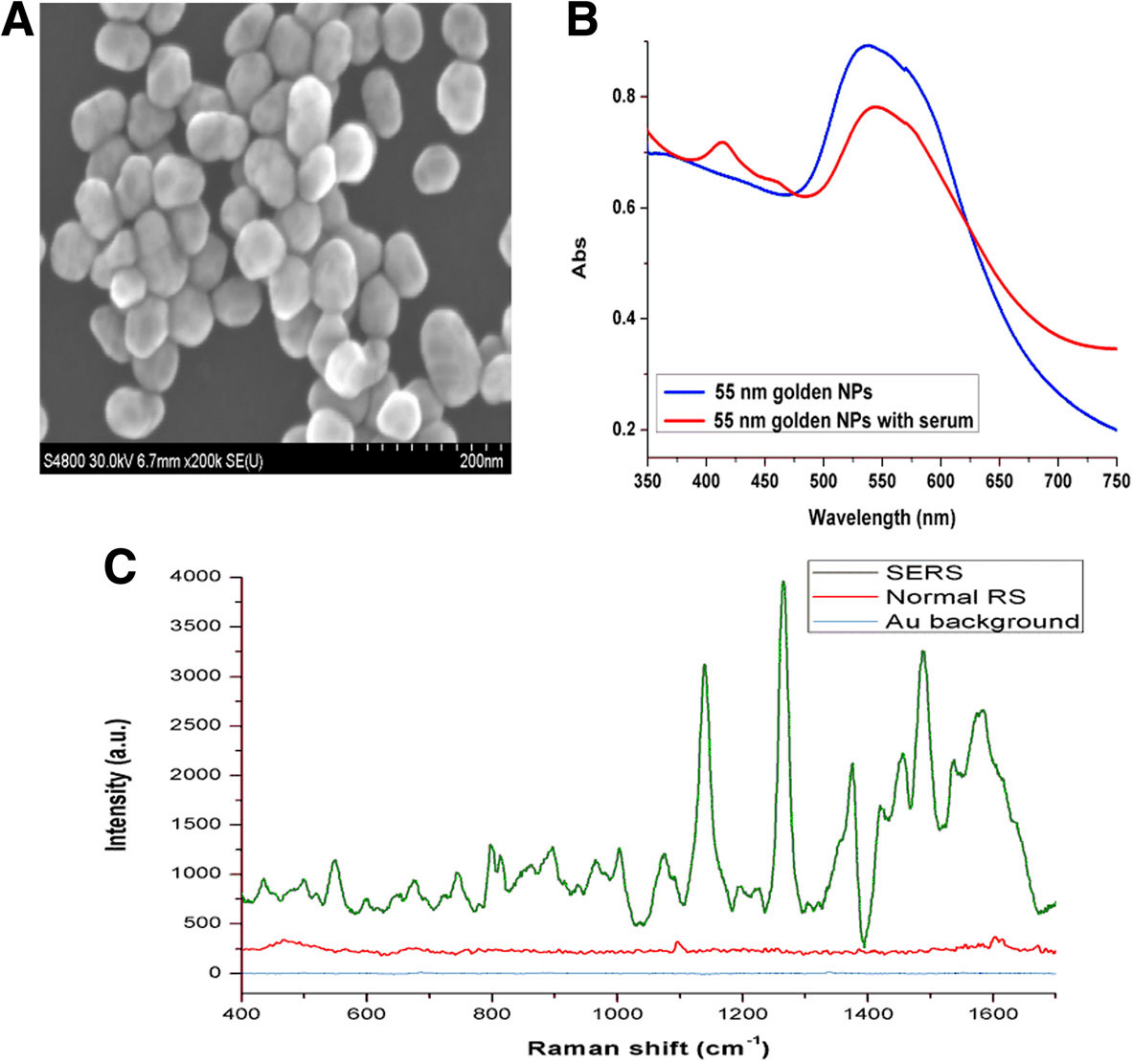
The SEM image, absorption curve and Raman spectrum of the gold NPs. **a** The SEM image of the gold NPs. **b** The UV/visible absorption of the gold NPs and the gold NPs with serum. **c** SERS spectrum of serum, routine Raman spectrum of serum and background Raman spectrum of the gold NPs

The SEM image of the pure gold NPs was shown in the Fig. 1a. The spherical NPs with a mean diameter of 55 nm were prepared in this method and had a maximal absorption at 530 nm. In the UV-visible absorption spectra shown in Fig. 1b, the pure NPs solution absorption band appeared in the around 530 nm wavelength region, and the band of the mixture of serum and NPs also remained in the same region but the intensity reduced due to the combination of gold NPs and biochemical substances in the serum. Compared with the regular Raman spectrum, the intensities of the SERS spectrum were enhanced enormously due to the gold NPs added in the serum (Fig. 1c).

**Fig. 2:**
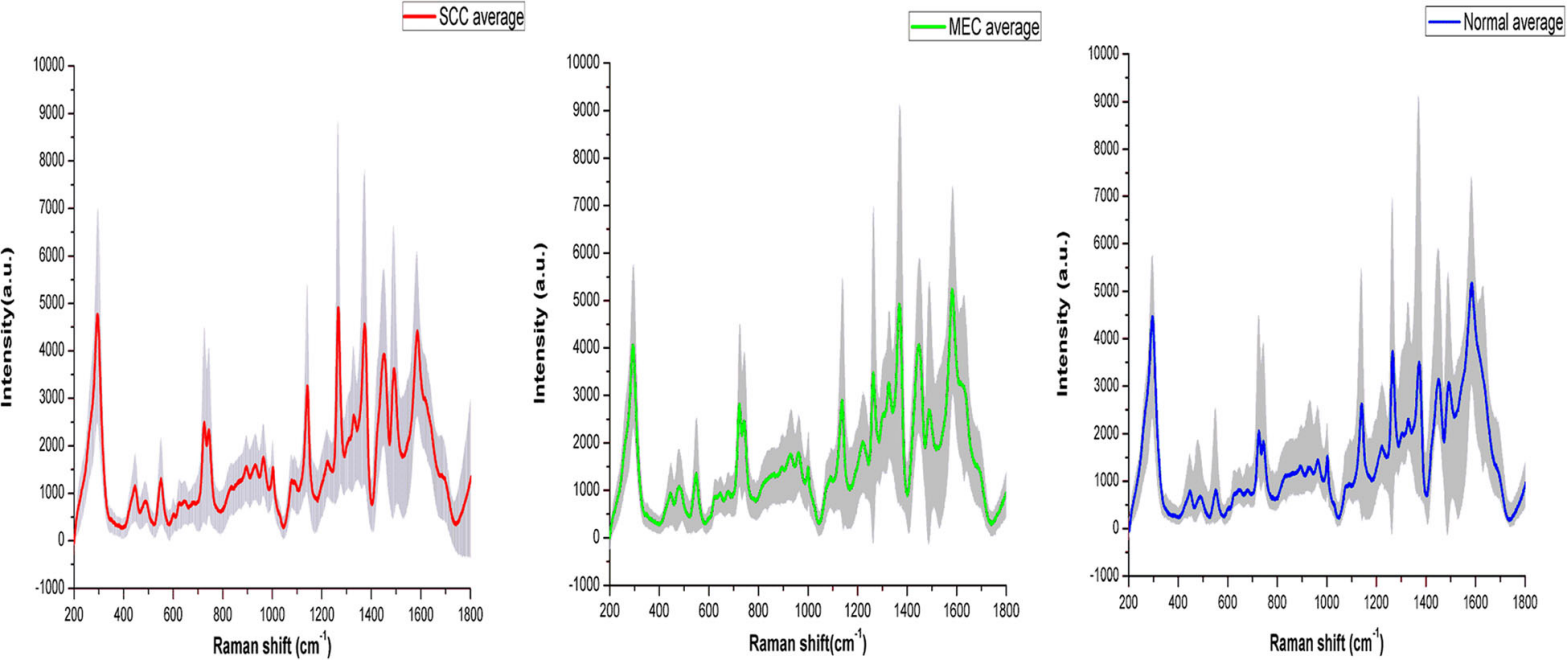
The average Raman spectra of OSCC, MEC and normal serum samples. The gray areas manifest the standard deviations

**Fig. 3:**
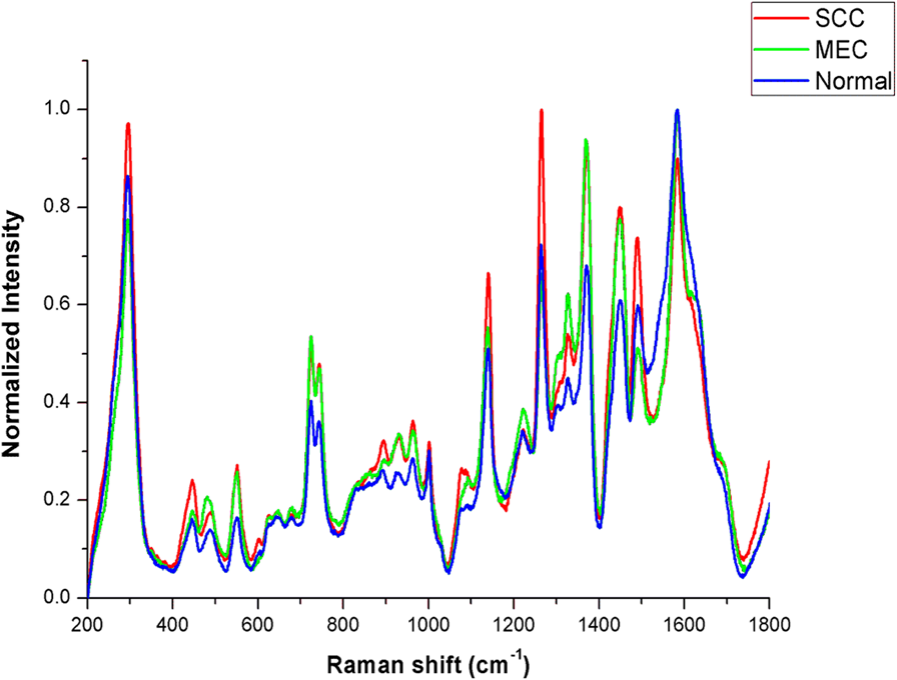
The normalized mean Raman spectra of OSCC, MEC and normal serum samples

**Fig. 4:**
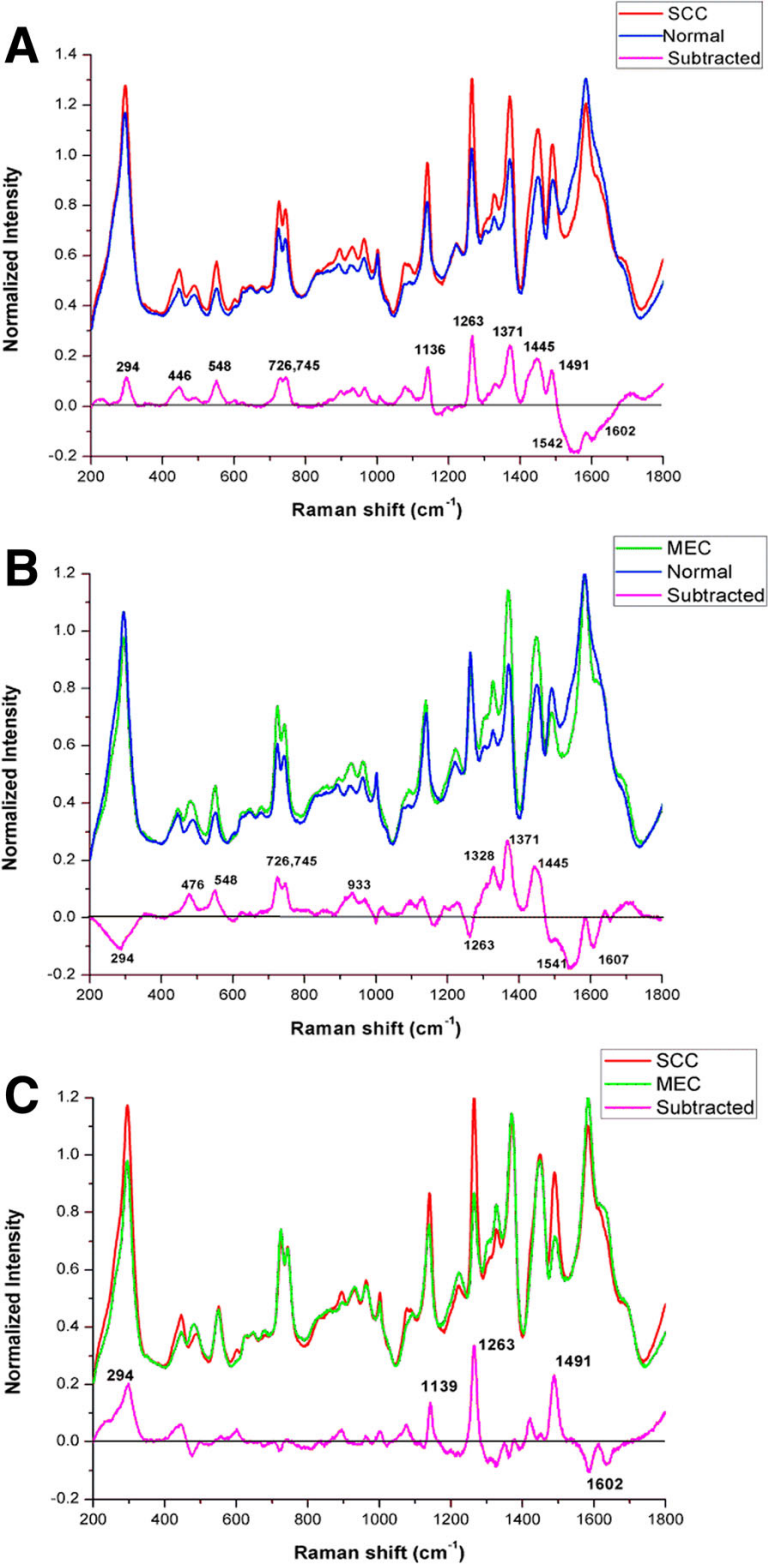
The subtracted spectra of the OSCC, MEC and normal serum samples

A total of 370 SERS spectra were recorded successfully in the Raman shift region from 200 cm^−1^ to 1800 cm^−1^. Among these spectra, 135 spectra were obtained from the clinically confirmed OSCC subjects, 90 spectra were obtained from the clinically confirmed MEC subjects and 145 spectra were obtained from the normal control subjects. The clinical data of the subjects participating in this study was shown in the Table 1. The mean spectra of different groups before the spectral normalization were presented in the Fig. 2. And after the spectral normalization, the normalized mean spectra were used to compare the spectral differences of the OSCC, MEC and normal groups (Fig. 3). Compared with the mean spectrum of the normal control group, the OSCC group showed the increase in the peaks at 294, 446, 548, 726, 745, 1136, 1263, 1371, 1445 and 1491 cm^−1^ but the decrease in the peaks at 1542 and 1602 cm^−1^, which were shown in the subtracted spectrum (Fig. 4a). Compared with the mean spectrum of the normal control group, the MEC group showed the increase in the peaks at 476, 548, 726, 745, 933, 1328, 1371 and 1445 cm^−1^ but the decrease in the peaks at 294, 1263, 1541 and 1607 cm^−1^ (Fig. 4b). The spectral differences were also presented in the subtracted spectrum between the OSCC group and MEC group. The subtracted spectrum showed the increase in the peaks at 294,1139,1263 and 1491 cm^−1^ but the decrease in the peaks at 1602 cm^−1^ in the OSCC group compared with the MEC group (Fig. 4c).

All these peaks of different intensities shown in the subtracted spectrum can be assigned to the various biochemical substances and molecular structures according to the reported literatures and previous studies [9, 13–18] (Table 2). Compared with the normal groups, the major increased peaks in the OSCC and MEC groups were assigned to the molecular structures of the nucleic acids and proteins. And these different major peaks between the OSCC and MEC groups were assigned to the special molecular structures of the carotenoids and lipids. The diagnostic classification of the different spectra can be carried out based on these peaks reported above.

**Table 2:** Raman shifts of peaks and the characteristic assignments

Raman shift (cm^−1^)	Peak assignment
294	Au-S band
446–476	Cholesterol
548	S-S disulfide stretching in Proteins
726	Hypoxanthine
745	Thymine in DNA
933	C-C stretching mode, C-C αhelix in proteins
1136–1139	C-N stretch in D-mannons
1263	CH bending in lipids
1328	CH vibration in DNA/RNA, CH_2_ twisting in lipids
1371	Guanine in DNA, Tryptophan in proteins
1445	CH_2_, CH_3_ bending in proteins and lipids
1491	CH_2_ bending
1541–1542	C-N stretching, Amide II
1602–1607	C = C band in Phenylalanine or Tyrosine

**Fig. 5:**
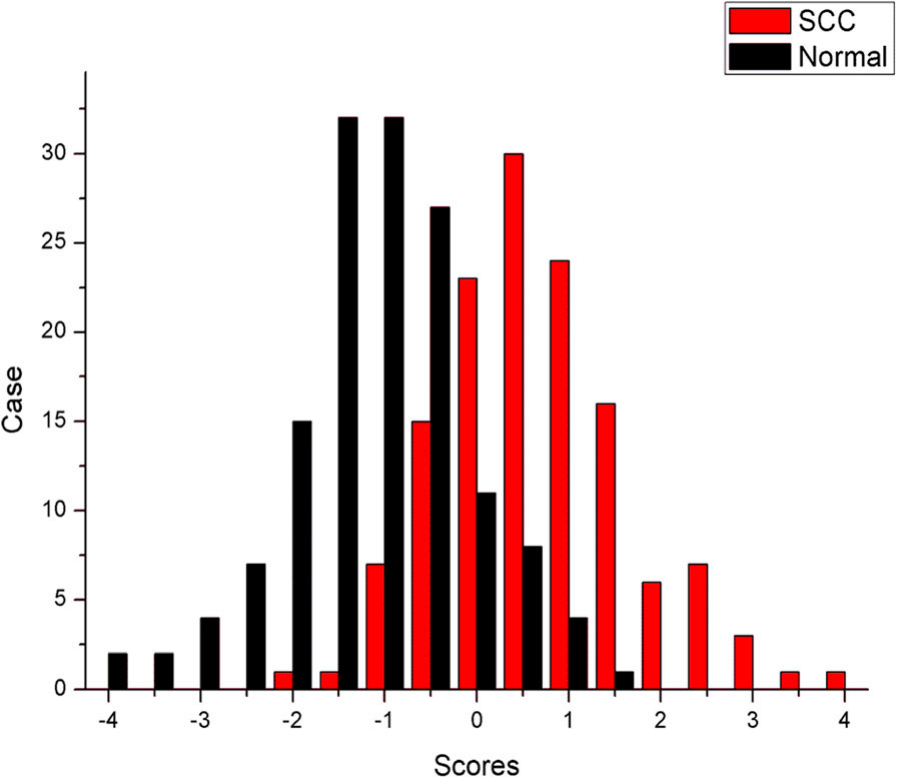
The histogram of discrimination scores of OSCC and normal group

In the first analytical step to discriminate the OSCC spectra from the normal ones, the OSCC spectra were selected as the positive group and the normal ones were selected as the negative group. The process of PCA extracted 55 principle components(PCs) from the raw spectral data, which captured about 95% of the cumulative variance of the raw data and were input as variables for the LDA process. As the result of the LDA, 109 of 135 OSCC spectra and 122 of 145 normal spectra were classified into the accurate group successfully (Table 3). The sensitivity and specificity were 80.7 and 84.1% respectively, and the total accuracy of this diagnostic classification was 82.5%. The histogram of discrimination scores demonstrated a clear classification of the two groups (Fig. 5). In order to test the results of the classification, the ‘leave-one-out’ method was employed in the cross validation process. And the result of the cross validation shown that 107/135 of OSCC spectra and 120/145 of normal spectra were diagnosed correctly (Table 4). The sensitivity and specificity of the diagnosis were 79.3 and 82.8%, and the total accuracy was 81.1%.

**Table 3:** The results of the classification of OSCC and normal group

Class	Predicted group			Total
		OSCC	Normal	
Count(%)	OSCC	109(80.7%)	26(19.3%)	135(100%)
	Normal	23(15.9%)	122(84.1%)	145(100%)

**Table 4:** The results of the cross validation of OSCC and normal group

Class	Predicted group			Total
		OSCC	Normal	
Count(%)	OSCC	107(79.3%)	28(20.7%)	135(100%)
	Normal	25(17.2%)	120(82.8%)	145(100%)

**Fig. 6:**
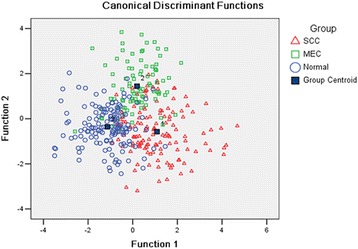
The scatter plot of discrimination scores of OSCC, MEC and normal group

In order to demonstrate the potential more effectively to diagnose the OSCC by SERS, the MEC spectra were selected as the positive control group in the diagnostic classification. The PCA process also extracted 51 PCs from the raw data, which accounted for about 93% of the total variance. Then the PCs were input in the LDA process to classify the three different groups. The result shown that 101/135 of OSCC spectra, 72/90 of MEC spectra and 129/145 of normal spectra were classified into the correct groups successfully (Table 5). The sensitivity and specificity of the diagnosis of OSCC were 74.8 and 89.0%, and the total accuracy was 81.6%. The scatter plot diagram showed the separation of the three groups (Fig. 6). Then the ‘leave-one-out’ method was employed in the cross validation process to test the results of the classification. In the results of the cross validation, 82/135 of OSCC spectra were classified correctly, 64/90 of MEC spectra and 109/145 of normal spectra were also diagnosed correctly. The sensitivity and specificity of the diagnosis of OSCC were 60.7 and 75.2%, and the total accuracy was 68.9% (Table 6).

**Table 5:** The results of the classification of OSCC, MEC and normal group

Class		Predicted group			Total
		OSCC	MEC	Normal	
Count(%)	OSCC	101(74.8%)	19(14.1%)	15(11.1%)	135(100%)
	MEC	3(3.3%)	72(80.0%)	15(16.7%)	90(100%)
	Normal	8(5.5%)	8(5.5%)	129(89.0%)	145(100%)

**Table 6:** The results of the cross validation of OSCC, MEC and normal group

Class		Predicted group			Total
		OSCC	MEC	Normal	
Count(%)	OSCC	82(60.7%)	27(20.0%)	26(19.3%)	135(100%)
	MEC	9(10.0%)	64(71.1%)	17(18.9%)	90(100%)
	Normal	19(13.1%)	17(11.7%)	109(75.2%)	145(100%)

## Discussion

The American Academy of Oral Medicine recognized that the patients with oral cancer detected in the early stages required less aggressive treatment and experienced fewer complications than the ones with advanced stage cancers, and the patients with early stage oral cancer would have improved survival [19]. And the evidence existed in a systematic review that the development and use of biomarkers was becoming increasingly common in the early detection of oral cancer [20]. The metabolites in the biofluids of the patients with cancers are different from the normal subjects, due to the amino acid metabolism, cell apoptosis and tumor necrosis, which can be used for the cancer detection as the biomarkers [10, 12]. The serum or plasma have been selected as the samples for the cancer detection and diagnosis by Raman spectroscopy successfully because the serum or plasma is easily collected and mostly contains some vital biomarkers generated by cancers [10, 15, 21, 22]. Rekha et al. [21] reported that the plasma Raman spectra of the oral premalignant and malignant conditions were different from the normal condition, and could be utilized to diagnose the oral premalignant and malignant conditions with the sensitivity and specificity of above 80.0%. Serum Raman spectroscopy had the potential not only in the diagnosis of oral cancers, but also in the prediction of treatment responses [22]. Accordingly, the serum was the sample of the choice in this study. The SERS has been employed to detect and diagnose the cancers with a high accuracy in different sites. High-quality SERS of serum was used to identify the colorectal cancer with the diagnostic accuracy of 100% [15]. The SERS in this study successfully made the serum constituents adsorb on the nanoparticles and captured the biomolecular spectra fingerprints of the biomarkers.

In this study, the spectral differences of the peaks assigned to various biomolecular fingerprints were captured in the SERS. Compared with the normal control group, the spectra of OSCC and MEC group exhibited the increasing intensities of the peaks at the 446 ~ 476, 548,726,745,1371 and 1445 cm^−1^. These peaks were assigned to the nucleic acids and proteins which could be caused by the active metabolism of nucleic acids in the patients with cancers [10, 22]. These findings were similar to the results reported in the previous studies. Rekha et al. [21] and Sahu et al. [22] all found that the increasing intensities of peaks at 1339 and 1445 cm^−1^ were related to the increase of the nucleic acids and proteins in the plasma and serum of the oral cancer groups. But in their reported studies, the ranges of Raman shifts were limited from the 700 cm^−1^ or 800 cm^−1^ to 180 cm^−1^, and the only routine Raman spectra were obtained, so there were some differences between their results and ours. Feng et al. [23] reported that the SERS band at 725 cm^−1^ were greater in nasopharyngeal cancer plasma than the ones in the normal plasma, and the band was assigned to the nucleic acids. The increasing proteins in the serum of OSCC and MEC could result from the redistribution or translocation of free amino acids in the blood of the patients with cancers [24]. Compared with the MEC group, there were the increasing intensities of peaks at 1139, 1263,1491 cm^−1^ and the decreasing intensity of peak at 1602 cm^−1^ in the SERS of OSCC group. These differences demonstrated the higher D-mannos and lipids level in the OSCC group. The previous study reported that there was an alteration in plasma lipid constituents in cancer patients because the cancer cells would utilize lipids for new membrane biogenesis [25]. So all above spectral differences captured in the SERS could be employed as the diagnostic indicators and references to detect the OSCC serum samples.

In order to develop and establish the classification and diagnostic models, numerous algorithms were employed to analyze Raman spectral data in the reported studies [21, 26–28]. Among these algorithms, PCA was a statistical technique for reducing the dimensions and simplifying complex data sets with the minimum reconstruction error [29]. However, PCA was not suitable for classification problems because it could not use any class information in computing the features extracted from the original data [30]. So Belhumeur et al. [31] combined PCA with LDA to make a popular method for dimension reduction and classification of data sets. LDA was a well-known method which found a linear transformation such that feature clusters were most seperable after the transformation [29]. The PCA combined with LDA were employed to classify and diagnose the different disease successfully based on the Raman spectral features [18, 23, 26, 32]. Rekha et al. [21] used PCA-LDA to yield a diagnostic sensitivity of 91.2% and a specificity of 96.7% in the classification of normal from oral malignant group. In this study, the PCA-LDA could also successfully classify and diagnose the spectra of OSCC and normal group with a sensitivity of 80.7% and a specificity of 84.1%. The results of the cross validation demonstrated the utilization of the PCA-LDA algorithm in the analysis of Raman spectral data was reliable. Then PCA-LDA had an excellent performance in the analysis and classification of the spectra of the OSCC, MEC and normal groups with a total accuracy of 68.9%, which showed a giant potential to diagnose OSCC based on the SERS.

## Conclusion

The gold NPs were appropriate substances to capture the high-quality surface enhanced Raman spectra of the OSCC, MEC and normal serum samples. In these Raman spectra, there were differences assigned to some specific molecular structures and components, which reflect the different levels of nucleic acids, proteins and lipids in the cancers and normal serums. These differences might result from the metabolic alterations, cell proliferation and apoptosis in the cancer serum. Based on these spectral differences and features, PCA-LDA could classify and diagnose the OSCC, MEC and normal groups successfully. In a conclusion, SERS combined PCA-LDA had a giant capability to detect and diagnosis OSCC through the serum sample successfully.

## Abbreviations

LDA: linear discriminant analysis
MEC: mucoepidermoid carcinoma
NPs: Gold nanoparticles
OSCC: oral squamous cell carcinoma
PCA: principal component analysis
SEM: scanning electron microscopy.
SERS: surface enhanced Raman spectroscopy
