# Correction to: Surface-enhanced Raman spectroscopy of blood serum based on gold nanoparticles for the diagnosis of the oral squamous cell carcinoma

**DOI:** 10.1186/s12944-019-0959-x

**Published:** 2019-02-13

**Authors:** Yingyun Tan, Bing Yan, Lili Xue, Yi Li, Xianyang Luo, Ping Ji

**Affiliations:** 1 Stomatological Hospital of Chongqing Medical University, Chongqing, 400000 China; 2 Chongqing Key Laboratory of Oral Diseases and Biomedical Sciences, Chongqing, 400000 China; 3 Chongqing Municipal Key Laboratory of Oral Biomedical Engineering of Higher Education, Chongqing, 400000 China; 4 Department of Otolarygology Head and Neck Surgery, the First Affiliated Hospital of Xiamen University, Xiamen, 361000 China; 5 Department of Stomatology, the First Affiliated Hospital of Xiamen University, Xiamen, 361000 China; 6 Department of Head and Neck Oncology, the West China Hospital of Stomatology, Sichuan University, Chengdu, 610000 China

**Correction to: Lipids in Health and Disease 2017 16:73**

**https://doi.org/10.1186/s12944-017-0465-y**

Following publication of the original article [1], the authors reported an error on the department of Funding in their paper. The Grant number of the National Science Foundation of China should be corrected from 81172578 to 81502584.
